# Allosteric Modulators for mGlu Receptors

**DOI:** 10.2174/157015907781695900

**Published:** 2007-09

**Authors:** F Gasparini, W Spooren

**Affiliations:** Novartis Institutes for BioMedical Research Basel, Neuroscience Research, WSJ-386.743, Postfach, CH-4002 Basel, Switzerland; F. Hofmann-La Roche, CNS Research, Psychiatry Disease Area, Building 74-148, 4070 Basel, Switzerland

## Abstract

The metabotropic glutamate receptor family comprises eight subtypes (mGlu1-8) of G-protein coupled receptors. mGlu receptors have a large extracellular domain which acts as recognition domain for the natural agonist glutamate. In contrast to the ionotropic glutamate receptors which mediate the fast excitatory neurotransmission, mGlu receptors have been shown to play a more modulatory role and have been proposed as alternative targets for pharmacological interventions. The potential use of mGluRs as drug targets for various nervous system pathologies such as anxiety, depression, schizophrenia, pain or Parkinson’s disease has triggered an intense search for subtype selective modulators and resulted in the identification of numerous novel pharmacological agents capable to modulate the receptor activity through an interaction at an allosteric site located in the transmembrane domain. The present review presents the most recent developments in the identification and the characterization of allosteric modulators for the mGlu receptors.

## INTRODUCTION

Since their identification in the mid eighties [56] and their subsequent cloning [18,31] in the early nineties, the metabotropic glutamate receptor (mGluR) family triggered an intensive search toward the identification of pharmacological agents to determine the role of the mGlu receptors in the nervous system physiology and patho-physiology. To date eight of such receptors (mGlu1-8) have been cloned and based on their amino-acids sequence, pharmacology and second messenger coupling these receptors have been clustered into three groups (I - III) [4]. mGlu receptors belong to the family 3 GPCRs and as such have a large extracellular ligand recognition N-terminal domain and seven transmembrane-spanning regions that are connected by three intra-cellular and three extracellular loops with a number of conserved cysteine residues [16]. The extracellular ligand recognition N-terminal domain has a so-called bi-lobed structure (= Venus fly-trap function) that can adopt an open or closed configuration in the absence or presence of an agonist, respectively [41]. In addition, there are indications that in the brain functional mGlu1 and mGlu5 receptors may predominantly exist as homodimers [52]. Glutamate is the physiological agonist for all mGlu receptors subtypes and as such the first selective agents identified were glutamate analogs [54] and are acting at the receptor by displacing Glutamate from its binding site located in the N-terminal domain, the orthosteric site. As such those ligands are named competitive agonists or antagonists. The structural analogy to Glutamate, with the presence of a distal carboxylic acid to the amino-acid functionality, strongly influence the properties of the competitive ligands and limits considerably their capacity to cross membranes by passive diffusion. As a consequence, these compounds have a poor oral bioavailability, and do not readily cross the blood-brain-barrier. A noticeable exception is illustrated by the small molecular weight ligands, LY354740, LY379268 or LY404039 [53] which display potent agonist activity at mGlu2/3 receptors, are orally bioavailable, and enter the brain in preclinical model [36,37] and have been developed throughout the clinic as potential anti-panic agents [2].

The difficulty to identify suitable glutamate analog with adequate drug-like properties and subtype selectivity has been an important element that fostered the search for alternative structural compound classes to inhibit or activate mGlu receptor function. 

## DISCOVERY

**Fig. (1):**
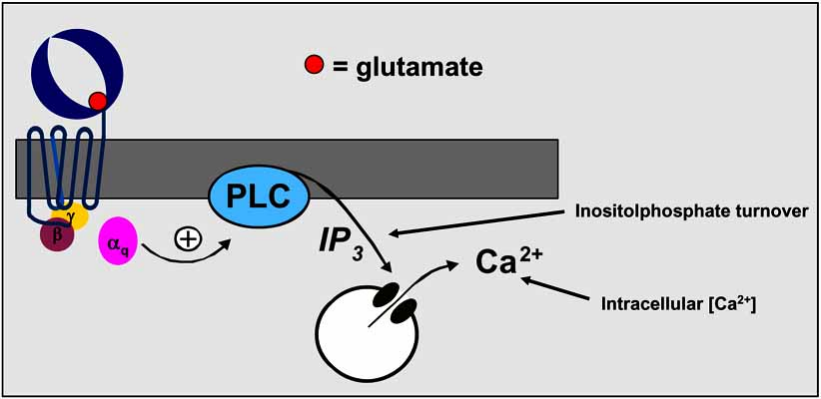
Group I mGluRs signal transduction pathway.

The identification of the allosteric modulators is intimately linked with the development of *in vitro* assays allowing the characterization of the functional activity of agents acting at the receptor. For the Group I mGlu receptors, coupled to Gq type of G-proteins, two cellular assays based on IP_3_ turnover and measurements of intracellular Ca^2+^ concentration were developed (Fig. **1**).

**Fig. (2):**
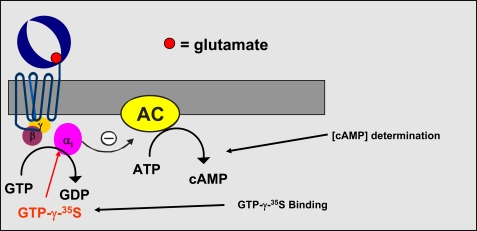
Group II and III mGluR signal transduction pathway.

For the Group II and III mGlu receptors, which are coupled to Gi type of G-proteins, functional assays involving GTP-γ-^35^S binding and determination of cAMP concentration changes were developed (Fig. **2**).

The development of these functional assays and their adaptation to a high throughput screening mode allowed the screening of large chemical libraries and the identification of numerous ligands with no structural analogies to the natural ligand Glutamate acting as negative allosteric modulators (NAM) or as positive allosteric modulators (PAM). 

A number of excellent reviews describing in the mechanism of action and the progresses in the allosteric modulator field have been published [13,14,20,44,45,50,55]. Here, we would like to describe the most recent developments in the identification and characterization of allosteric ligands for the various mGluR subtypes. 

## GROUP I 

### mGluR1 NAM

CPCCOEt (**1**) was the first non glutamate antagonist described for the mGlu1 receptor [1]. This agent has been a useful tool to investigate the role of the mGluR1 subtype *in vitro*, however, its limited potency (only low micromolar IC_50_), its lack of bio-availability and brain penetration did not allow a broad use *in vivo*. Nevertheless, CPCCOEt was identified as non-competitive inhibitor and allowed to unravel and characterize the allosteric binding site located in the transmembrane domain [28]. A more potent compound, BAY36-7620 (**2**) was subsequently described to interact at the same allosteric binding site and to inhibit the receptor constitutive activity [3]. The improved *in vivo* properties of BAY36-7620 has allowed the detection of an early signal for analgesic and anticonvulsant effect but also for an impaired acquisition of a spatial memory task (Morris water maze) [9,58]. This effect on memory was later confirmed with highly optimized compounds. However, various issues including potency has limited an extensive characterization using this compound for the use of exploring the potential of mGlu1 receptor antagonists for the treatment of various CNS disorders. The first described highly potent, selective and systemically active mGlu1 receptor antagonist is JNJ16259685 (**3**) [25]. JNJ16259685 is a non competitive antagonist with IC_50_ values of 3.2 and 1.2 nM in glutamate-induced calcium mobilization at rat and human receptors, respectively. In addition, systemic application of JNJ16259685 resulted in high receptor occupancy at low dosis. Together these data provide evidence that JNJ16259685 is an excellent tool to explore the potential of mGlu1 receptor antagonists as therapeutics [25]. Steckler *et al.* [60] described anxiolytic-like properties in punished licking behavior which appeared to have synergistic effects with mGlu5 receptor antagonists. Nevertheless, the effects seem different from those for mGlu5 receptor antagonists as prominent effects were only seen on conflict procedures but not on task based spontaneous exploration. Effects were described on locomotor activity as expected from the expression pattern in cerebellar structures but following chronic application data suggest tolerance to these motor disturbances. Subsequently, Steckler *et al.* [61] described that JNJ16259685 impaired spatial acquisition processes, irrespective of spatial load, as well as spatial re-acquisition, already at the lowest dose tested (0.63 mg/kg). Effects on spatial retention performance were relatively mild in mice that had learned to locate the position of the escape platform prior to treatment. These data suggest that blockade of the mGlu1 receptor primarily affects learning of new information, but leaves retention of spatial information relatively unaffected.

Another potent and selective non-competitive mGlu1 antagonist is A-841720 (**4**) [10]. At recombinant human and native rat mGluR1 receptors, A-841720 inhibited agonist-induced calcium mobilization, with IC_50_ values of 10.7 and 1.0 nM, respectively, while showing selectivity over other mGluR receptors. Intraperitoneal injection of A-841720 potently reduced complete Freund's adjuvant-induced inflammatory pain (ED_50_ = 8 mg/kg) and monoiodoacetate-induced joint pain (ED_50_ = 15 mg/kg). A-841720 also decreased mechanical allodynia observed in both the sciatic nerve chronic constriction injury and L5-L6 spinal nerve ligation (SNL) models of neuropathic pain (ED_50_ = 10 and 9 mg/kg, respectively). Electrophysiological studies demonstrated that systemic administration of A-841720 in SNL animals significantly reduced evoked firing in spinal wide dynamic range neurons. Importantly, significant motor side effects were observed at analgesic doses and A-841720 also impaired cognitive function in the Y-maze and the Water Maze tests with no therapeutic index. The analgesic effects of a selective mGluR1 receptor antagonist are associated with motor and cognitive side effects.

Taken together, the lack of separation between efficacy and side effects in pre-clinical models indicates that mGluR1 antagonism may not provide an adequate therapeutic window for the development of such antagonists as therapeutics in humans.

### mGluR1 PAM

**Fig. (3):**
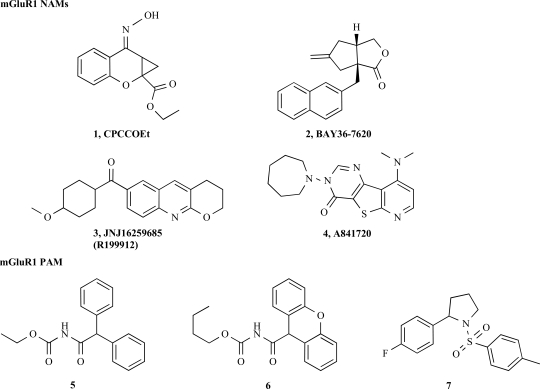
mGluR1 allosteric modulators.

Knoflach *et al.* [23] were the first to describe two chemical series, compounds **5**, **6** and **7** (Fig. **3**), capable to potently increase agonist-mediated responses in cells expressing the rat mGlu1 receptor and in rat brain tissues. All three compounds were shown to enhance the binding affinity of agonists for the orthosteric site. In cells recombinantely expressing high level of the receptor and in which the receptor shows a significant level of constitutive activity, all three compounds were found to elicit a response in the absence of orthosteric agonist. No similar effect was seen in native systems. From the mechanistically point of view these effects were explained by an interaction of the compound with an active form of the receptor and the stabilization thereof. Compounds **5** and **7**, showed selectivity over the rmGlu2, rmGlu4, rmGlu5, rmGlu8 and hGABA_B_ receptors whereas compound **6**displayed a weak activation of the glutamate-induced activation at the rmGlu5 receptor. Interestingly, when tested on the human mGlu1 receptor only compound **6 ** acted as positive modulator, compounds **5** and **7** being devoid of activity. Furthermore, using chimeric receptor constructs and site directed mutagenesis, Knoflach *et al.* [23], could demonstrate that all three compounds interact in the transmembrane domain in an overlapping pocket compared to the mGluR1 NAMS. Recently, a series of structurally different mGlu1 positive modulators obtained by chemical derivatization of the mGluR5 PAM CDPPB (**15**, Fig. **3**) was described and characterized. In contrast to the compounds **5**, **7** and **8**, the CDPPB series was shown to interact in the transmembrane domain with a different binding mode compared to the mGluR1 allosteric negative modulators [8].

So far, no *in vivo* activity has been reported on the effects of mGlu1 receptor PAMs, but based on the negative effects of NAMs on learning and memory [10,61] it can be hypothesized that enhancement of mGlu1 receptor activity through a selective PAM could be beneficial to restore normal learning and memory processes.

### mGluR5 NAM

2-methyl-6-(phenylethynyl)-pyridine (MPEP), **8** is the prototypical allosteric mGlu5 receptor antagonist [12]. It is a potent, selective and systemically active antagonist. At the human mGlu5a receptor expressed in recombinant cells, MPEP completely inhibits quisqualate-stimulated phosphoinositide (PI) hydrolysis with an IC_50_ value of 36 nM while having no agonist or antagonist activities at other receptors up to 30 μM. Schild's analysis indicated that MPEP acts in a non-competitive manner. MPEP also inhibited to a large extent constitutive receptor activity in cells transiently overexpressing rat mGluR5, suggesting that MPEP may act as an inverse agonist [40].

Using chimeras and single amino acid substitutions between hmGluR1 and hmGluR5, binding of the mGluR5 radioligand [^3^H]-2-methyl-6-(3-methoxyphenyl)ethynyl pyridine (M-MPEP) it could be determined that the amino acids Ala-810 (TMVII), Pro-655 and Ser-658 (TMIII) of the mGlu5 receptor are necessary to mediate the binding and the selectivity of the MPEP series of antagonists. Conversely, the introduction of these three amino-acids in the corresponding reciprocal position of the hmGlu1 receptor led to a mutant mGlu1 receptor capable to bind [^3^H]-M-MPEP with low nanomolar affinity [40]. Radioligand binding to these mutants was also inhibited by 7-hydroxyiminocyclopropan [b]chromen-1a-carboxylic acid ethyl ester (CPCCOEt), a structurally unrelated non-competitive mGluR1 antagonist previously shown to interact within the TM region of the mGlu1 receptor [28]. These results indicate that MPEP and CPCCOEt bind to overlapping binding pockets in the TM region of group I mGluRs but interact with different non-conserved residues. Several additional allosteric mGlu5 antagonists based on the chemical structure of MPEP have been described and characterized, among those MTEP **9** a close derivative was also extensively characterized [5,57].

The identification of MPEP (2-methyl-6-(phenylethynyl)-pyridine allowed the exploration of the therapeutic potential of this class of compounds. Subsequent behavior studies revealed that--with the exception of benzodiazepines--mGlu5 receptor antagonists exhibit the widest and most robust anxiolytic activity in preclinical models seen to date [59].

### Fenobam

Fenobam [N-(3-chlorophenyl)-N'-(4,5-dihydro-1-methyl-4-oxo-1H-imidazole-2-yl)urea] (**10**) is an atypical anxiolytic agent, with until recently an unknown molecular target, that has previously been demonstrated both in rodents and human to exert robust anxiolytic-like activity. During a HTS screen at F. Hoffmann-La Roche, Fenobam (McN-3377) was identified as a potent antagonist at mGlu5 receptors [49]. Characterization revealed that Fenobam is a selective and potent metabotropic glutamate mGlu5 receptor antagonist acting at an allosteric modulatory site shared with 2-methyl-6-phenylethynyl-pyridine (MPEP). Fenobam inhibited quisqualate-evoked intracellular calcium response mediated by human mGlu5 receptor with IC_50_ = 58 nM. It acted in a noncompetitive manner, similar to MPEP and demonstrated inverse agonist properties, blocking 66% of the mGlu5 receptor basal activity (in an over expressed cell line) with an IC_50_ = 84 nM. [^3^H]-Fenobam bound to rat and human recombinant receptors with K_*d*_ values of 54 and 31 nM, respectively. MPEP inhibited [^3^H]-fenobam binding to human mGlu5 receptors with a K*i* value of 6.7 nM, indicating a common binding site shared by both allosteric antagonists. Fenobam exhibits robust anxiolytic-like activity with efficacy comparable to MPEP in the stress-induced hyperthermia model, Vogel conflict test, Geller-Seifter conflict test, and conditioned emotional response with a minimum effective dose of 10 to 30 mg/kg p.o. Furthermore, Fenobam is devoid of GABAergic activity, confirming previous reports that Fenobam acts by a mechanism distinct from benzodiazepines. The non-GABAergic activity of Fenobam, coupled with its robust anxiolytic activity supports the potential of developing mGlu5 receptor antagonists with an improved therapeutic window over benzodiazepines as novel anxiolytic agents. The confidence in mGlu5 receptor antagonists as a treatment for anxiety is obviously strongly supported by positive results in clinical trials with patients. There are at least two positive trials that indicate that Fenobam is indeed efficacious in man including a double blind placebo-controlled and standardized (Diazepam) trial [42,43]. It was concluded from these studies that Fenobam relieved symptoms of anxiety in broad spectrum psychoneurotic patients which may experience anxiety as cognitive, dysphoric or motor autonomic. The anxiolytic activity is differentiated from benzodiazepines and fenobam was safe and induced less sedation and drowsiness. Clearly, these findings need to be confirmed in upcoming clinical trials using highly optimized mGlu5 receptor antagonists but provide great promise for future studies.

### mGluR5 PAM

In contrast to the high number of NAM for the mGlu5 receptor, relatively few positive allosteric modulators have been described. The first series of compounds is based on a benzaldazine core structure and the pharmacological activity is determined by the nature and the position of the substituents [38]. In this series, DFB (3,3’-difluorobenzaldazine, **11**, Fig. **5**), acts as positive modulator and induces a marked increase in glutamate potency (EC_50_). Interestingly, a change in the nature of the substituent with a replacement of the two fluorine atoms by a methoxy group conferred an antagonistic activity to the resulting DMeOB (3,3’-dimethoxyBenzaldazine, **12**, Fig. **5**) derivative. Another very subtle substitution change, chlorine for the fluorine atoms, led to the identification of a ligand with no functional activity at the receptor, DCB (3,3’-dichloro-benzaldazine, **13**, Fig. **5**). The DFB family of modulators is the first to demonstrate the whole range of pharmacological activities from activation to inactivation of the receptor as well as neutral/silent receptor ligands. All active derivatives of this series are interacting in the transmembrane domain in a similar but not identical binding site to the mGluR5 NAMs of the MPEP structural family. Shortly after, two novel, structurally unrelated series illustrated by the prototypic compounds CPPHA (**14**) and CDPPB (**15**) were described by the same research group [27,39]. Both compounds showed a potent increase of agonist mediated receptor activation for both human and rat receptors with no cross reactivity at the other mGluR subtypes nor at other CNS receptors. No activation of the receptor was seen when DFB or CPPHA were used in the absence of orthosteric agonist [39] whereas CDPPB alone was capable to induce a receptor activation [22]. All three compounds had no effect on the binding of the orthosteric ligand [^3^H]-quisqualate [22,39] whereas only DFB and CDPPB did inhibit the binding of [^3^H]-methoxy-PEP-y [5] a derivative of the allosteric negative modulator MPEP. Despite intense medicinal chemistry efforts, neither the DFB nor CPPHA series did not deliver suitable molecules for *in vivo* evaluation in preclinical models. In contrast, derivatization of the CDPPB series not only allowed the identification more potent derivatives but also the identification of mGluR1 positive allosteric modulators [8].

*In vivo*, CDPPB was evaluated in two behavioral models sensitive to antipsychotic: the amphetamine-induced locomotor activity and the amphetamine-induced disruption of prepulse inhibition (PPI) in rat. In both models, CDPPB showed a dose dependent beneficial effect without noticeable side-effects [22]. These results demonstrated for the first time the *in vivo* activity of a selective mGluR5 allosteric positive modulator and suggested a potential use of these type of modulator as anti-psychotics.

## GROUP II 

### mGluR2 NAM

**Fig. (4):**
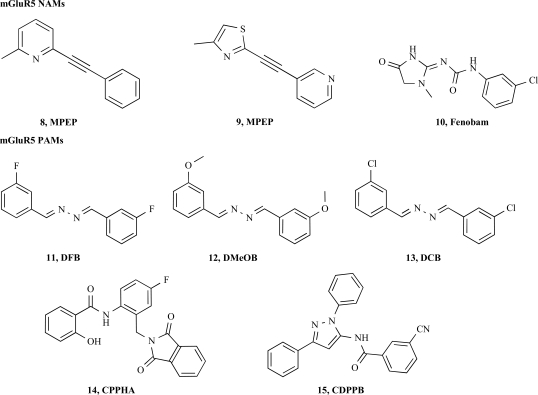
mGluR5 allosteric modulators.

Since the publication of two series of mGluR2 NAMs by Kolczewski *et al*. [24] and Wichmann *et al*. [63] no further development nor structurally novel mGluR2 NAMs have been described to date. These two series illustrated by the compounds **16** and **17** (Fig. **4**) were shown to inhibit agonist stimulated GTP-γ-[^35^S] binding on a rat mGluR2 transfected cell membranes with sub-micromolar IC_50_. Both lead compounds were also shown to be selective over mGluR1, mGluR4, mGluR5 and ionotropic GluRs but no data was published for the selectivity over the mGluR3 or other CNS receptors. Based on the effects achieved with orthosteric antagonists, it could be speculated that mGluR2 NAMs could improve cognitive and or memory disturbances [17].

### mGluR2 PAM

Among all mGlu receptor subtypes, the activation of the Group II subtypes is by far the best studied thanks to the identification and development by Eli Lilly of the orthosteric agonist LY354740 [36,37]. This compound and its prodrug derivative (LY544344) have been extensively characterized in preclinical model for anxiety related disorders and subsequently developed in the clinic where their use demonstrate the usefulness of activation of Group II receptors for the treatment of anxiety related disorders [26]. This proof of principle with LY354740 also confirms that activation of the mGluR2,3 subtypes display therapeutic activity at doses which are devoid of the side effects typically observed with agents interacting with the ionotropic glutamate receptors.

**Fig. (5):**
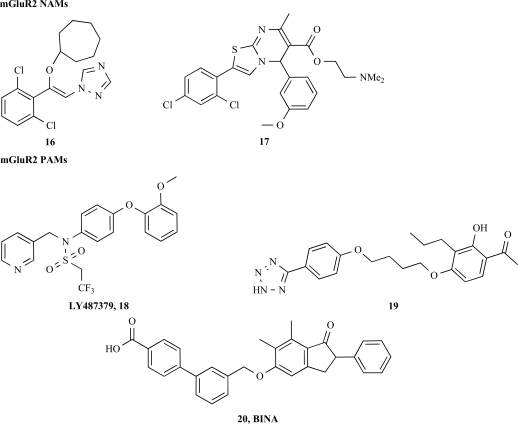
mGluR2 allosteric modulators.

The search for orthosteric ligands derived from the original structure, LY354740, with similar pharmacological and drug-like properties proved to be extremely difficult and prompted the search for alternative structures. As for the Group I, the use of functional assays combined with the screening of chemical libraries allowed the identification of the first series of positive allosteric modulators: the sulfonamide derivatives such as LY487379 (**18**) [19]. LY487379 and analog compounds from the same chemical class were shown to act as allosteric modulators with an activation of the receptor only in the presence of an orthosteric agonist and no displacement of the orthosteric antagonist LY341495. In contrast to all known orthosteric agonists, LY487379 and its derivatives are selective for the mGluR2 over the other seven mGluR subtypes including the mGluR3 and over a binding battery of CNS relevant receptor. *In vivo*, LY487379 or its derivatives demonstrated efficacy in animal models for anxiety (Fear potentiated startle; Stress induced hyperthermia) or for anti-psychotic activity (PCP induced locomotor activity) [21]. A second series of compound was described shortly after by a research group at Merck and exemplified by the phenyl-tetrazolyl acetophenone derivative **19** (Fig. **5**) [7,46,47,48]. *In vitro*, this series of compounds showed a similar profile to the LY487379 and *in vivo* the use of a more potent and brain penetrable compound, BINA (**20**), confirmed the potential anti-psychotic effects in animal models [11,15,48,51].

Taken together these results that mGluR2 selective modulators constitute a potential alternative for a pharmacotherapeutic intervention to the orthosteric non-selective mGluR2,-3 agonists. 

## GROUP III 

### mGluR4, 6, 7, 8 NAMs

Compared to the Group I and II mGluR, the pharmacology of the Group III is the least developed with a lack of subtype selective negative modulators. To our knowledge, only one series of compounds claimed in a patent (WO02102807; EP1408042 A1) has been described. Within this series compound **23** is described as a selective allosteric negative modulator of the mGluR7 with an IC_50_ of 7 nM.

### mGluR4 PAM

For the mGlu4 receptor, two compounds have been identified, PHCCC, **21** [29] and SIB1893, **22** [33] both originally discovered as antagonist for the mGlu1 and mGlu5 receptors, respectively. MPEP, **8** was also identified as potentiator of the mGluR4 but at very high concentrations (50-100 μ m) which are several order of magnitude higher than the effective concentrations needed to inhibit the mGlu5 receptor. 

In rat brain slices, using CPCCC Valenti *et al.* [62] could demonstrate that the mGlu4 receptor plays a key role in the Group III mGluR modulation of the excitatory transmission in the substantia nigra pars compacta (SNc), a key region involved in the nigral neurodegeneration and the resulting movement disorders in Parkinson’s Disease. Unfortunately, the limiting drug-like properties of PHCCC and the lack of more potent and selective mGluR4 PAM prevent the validation *in vivo* in animal model of this putative use of mGluR4 positive modulators. Additional potential pharmacotherapeutic uses of mGluR4 modulators have been recently reviewed by Marino *et al.* [30].

### mGluR7 PAM

As for the other members of the Group III receptors the lack of suitable selective pharmacological tools slowed considerably the study of the mGlu7 receptor. However, the generation and the characterization of the mGluR7 knock-out mice indicated that amygdala dependent functions such as conditioned fear, aversive responses and responses to anxiety and stress related responses are altered [6,32,35].

**Fig. (6):**
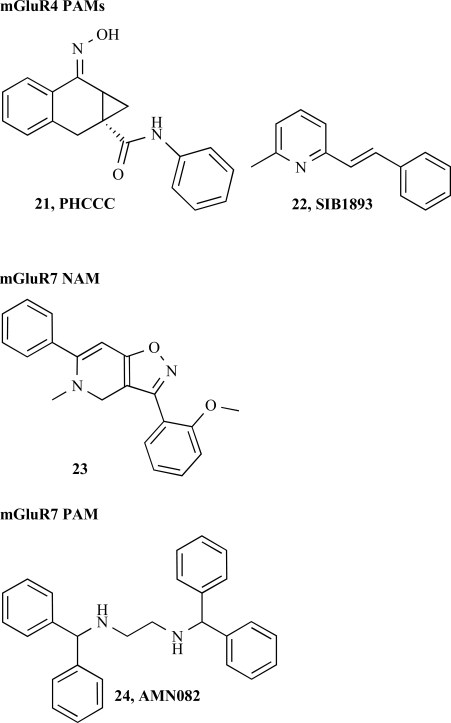
Group III allosteric modulators.

The recent identification of a selective allosteric agonist, AMN082 (**24**, Fig. **6**) allowed the confirmation of the data obtained with the mGluR7 null mice [34]. *In vitro*, AMN082 was shown to potently activate the recombinantly expressed human mGlu7 receptor with an EC_50_ of 64 nM in the absence of orthosteric agonist. In addition, AMN082 increases the orthosteric agonist (Glu, L-AP4) induced receptor activation in analogy to the positive modulators at the other mGluR subtypes. *In vivo*, AMN082 was shown to be orally bioavailable, to penetrate into the brain and to modulate the level of stress hormones (cortisol and ACTH) in wild type animal but not in the mGluR7 ko mouse. Taken together this data demonstrate that mGluR7 modulation could a valuable mechanism for the pharmacotherapy of stress-related psychiatric disorders.

## CONCLUSIONS/OUTLOOK

The ubiquitous distribution of glutamate in the brain sets off the critical role of glutamate receptors in most major functions of the CNS. Next to the ionotropic glutamate-gated ion channels, metabotropic glutamate receptors modulate neuronal excitability, synaptic transmission, and have various metabolic functions. Albeit small, the Family III or Class C of G-protein-coupled receptors have become the focal point for the discovery of new and exciting modulators of glutamatergic neurotransmission. Exciting medicinal chemistry that allowed the interaction of small molecules with receptors through soft touched allosteric modulation that modify transmembrane signaling rather than compete for binding with the natural agonist at orthosteric sites. Indeed, the mGlu receptors are illustrative in the discovery of both positive and/or negative allosteric modulators that display a unique degree of subtype selectivity within the highly conserved mGlu family of receptors. The combination of these pharmacological tools, in conjunction with genetic approaches, has led to major advances in our understanding of the roles of mGlu receptors in regulating of CNS function and animal behavior. From cloning to the identification multiple receptors, sub-type selective allosteric modulators and testing in double blind placebo controlled trials in less than 15 years is a remarkable story. These developments provide us with the unique opportunity for better treatments in a wide variety of neurological and psychiatric disorders that include complicated disease states such as depression, anxiety disorders, and schizophrenia.
